# The effect of an antioxidant gel compared to chlorhexidine during the soft tissue healing process: An animal study

**DOI:** 10.1002/JPER.23-0794

**Published:** 2024-06-03

**Authors:** Kelechi Ukaegbu, Deborah Foyle, Xianghong Luan, Emet Schneiderman, Edward P. Allen, Jacqueline Plemons, Kathy K. H. Svoboda

**Affiliations:** ^1^ Department of Periodontology Texas A&M School of Dentistry Dallas Texas USA; ^2^ Department of Biomedical Sciences Texas A&M School of Dentistry Dallas Texas USA; ^3^ Department of Oral and Craniofacial Sciences University of Rochester Rochester New York USA

**Keywords:** antioxidants, chlorhexidine, oxidative stress, wound healing

## Abstract

**Background:**

Prolonged inflammation and oxidative stress can impede healing. To enhance healing efficiency, many solutions have been employed. This is an in vivo study comparing chlorhexidine (CHX) to a commercial antioxidant gel (AO).

**Methods:**

Envelope flaps were created in the lower incisor gingival region of 60 Sprague–Dawley rats, and acellular dermal matrix (ADM) was inserted. Animals were randomly assigned to postsurgical treatment application of AO gel or 0.12% CHX twice daily. A control group received no postsurgical treatment. Data collected (before surgery, 24 h, and 72 h) included surgical images, tissue samples, and weights. Blinded scorers assessed images using a wound healing scale. Real‐time polymerase chain reaction (RT‐PCR) was used for gene expression of tumor necrosis factor‐alpha (TNFα), interleukin‐1 (IL‐1), myeloperoxidase (MPO), and superoxide dismutase (SOD).

**Results:**

The AO group scored higher than the CHX and control groups in clinical evaluation (*p* < 0.05). At 24 h, TNFα expression was upregulated in the AO group compared to CHX (*p* = 0.027) and controls (*p* = 0.018). The AO group had significantly higher expression of antioxidant enzyme (SOD) at 24 h compared to CHX (*p* = 0.021). All animals lost weight in the first 24 h. Animals treated with AO or CHX regained more weight at 72 h than control animals (*p* = 0.034 and 0.003, respectively).

**Conclusion:**

Animals treated with AO healed faster. AO led to earlier upregulation of TNFα and antioxidant enzyme SOD. We hypothesized that AO promoted an earlier inflammatory process while counteracting oxidative stress by increasing antioxidant responses via SOD.

## 1 INTRODUCTION

Mucogingival defects, characterized by inadequate keratinized tissue, reduced gingival thickness, and gingival recession, represent a challenge in adult periodontal care.^1^, ^2^ The incidence of these defects increases with age and occurs across populations with varying oral hygiene standards.^3^, ^4^ The prevalence of these defects underscores the need for effective therapeutic strategies. Rehabilitating mucogingival defects has become a cornerstone of modern periodontal therapy, as the presence of healthy soft tissue architecture surrounding teeth and implants is integral to long‐term functional and aesthetic success.^5^ Conventionally, free gingival grafts and connective tissue grafts have been used to increase attached gingiva and improve root coverage. Some patients prefer non‐autogenous soft tissue grafting materials due to their palate‐free technique such as xenogeneic collagen matrix (XCM), synthetics, and acellular dermal matrix (ADM) which allow the simultaneous management of multiple defects and provide a uniform and supportive matrix for tissue regeneration.^6^, ^7^, ^8^

Despite the advancements in surgical techniques and biomaterials, the complex wound healing process remains vulnerable to oxidative stress that arises from an imbalance between reactive oxygen species (ROS) and antioxidant defenses. Briefly, following injury and hemostasis, inflammation ensues, characterized by swelling and pain. Inflammatory cytokines tumor necrosis factor (TNFα) and interleukin‐1 (IL‐1) are released recruiting leucocytes to the site. ROS are generated from neutrophils and macrophages which contain NADPH oxidase, an enzyme that converts oxygen to a superoxide radical (O_2_^−^). Myeloperoxidase (MPO) combines H_2_O_2_ with chloride to form hypochlorous acid (HClO), another ROS.^8^ Superoxide dismutase (SOD) is an antioxidant enzyme that catalyzes the conversion of O_2_^−^ into O_2_ and hydrogen peroxide (H_2_O_2_). ROS play a dual role in wound healing, functioning as essential signaling molecules for processes like chemotaxis and cell proliferation while simultaneously impeding repair through oxidative damage to cellular components.^10^ This intricate balance between ROS and antioxidants highlights the need to regulate their levels to ensure effective wound healing.

Antioxidants are defined as “any substance that, when present at low concentration compared with those of an oxidizable substrate, significantly delays or prevents oxidation of that substrate”^11^ and play a vital role in modulating oxidative stress. Endogenous enzymatic antioxidants, for example, SOD, work in tandem with nonenzymatic exogenous antioxidants such as vitamins A, C, and E, carotenoids, polyphenols, and flavonoids to maintain redox homeostasis.^12^ Both have garnered attention for their potential to mitigate oxidative stress and enhance wound healing outcomes.

In the context of post‐mucogingival surgical wound healing, chlorhexidine (CHX) has been widely employed as an antimicrobial adjunct to mechanical oral hygiene regimes. Its cationic nature allows it to interact with bacterial cell membranes, disrupting membrane integrity and exerting antibacterial effects. CHX has high substantivity, enabling its release over 8–12 h, which is beneficial in inhibiting pellicle and plaque formation.^13^ While CHX effectively combats oral pathogens, it has drawbacks, including oxidative stress induction.^14^ In addition, CHX negatively affects fibroblast function and collagen synthesis, which are crucial for wound healing^15^ and can cause temporary taste disturbance and tooth staining.^16^

An antioxidant gel (AO) has demonstrated the ability to reduce gingival inflammation in a clinical study.^17^ It is composed of ingredients including antimicrobial agents (menthol, thymol, xylitol) and antioxidant flavonoids (phloretin and ferulic acid). The latter combination has been found to counter adverse effects on oral fibroblasts triggered by ROS, offering multifaceted defense mechanisms.^18^ The flavonoids exhibit diverse actions, encompassing radical scavenging, and pathogen inactivation.^19^ The gel is currently being used following soft tissue procedures.

To date, there is a lack of in vivo and clinical studies comparing antioxidant agents to the current gold standard (CHX) following periodontal intervention. This in vivo study investigates the impact of antioxidants on post‐mucogingival surgical wound healing, comparing the effects of an AO with CHX treatment using a rodent model. Animals serve as translatable^20^ models for investigating wound healing post periodontal surgery.^21^, ^22^ The similarity between the dentogingival structures of rats and humans supports the use of rats for investigating healing.^23^ Additionally, pain and the progression of wound healing can be monitored.^24^, ^25^

The primary aim of this study was to compare postsurgical clinical outcomes of the topical application of CHX with AO. To support the clinical outcome, the effect of CHX and AO on physical and behavioral outcomes including gene expression of acute inflammation and antioxidant markers, as well as pain, and the effect on normal feeding behavior measured by monitoring weight change were documented.

## 2 MATERIALS AND METHODS

### 2.1 Study design, ethical approval, and setting

The experimental model used in this study involved placing ADM* in an envelope flap confined to the mandibular gingival tissues using a previously established rat model, where a significant difference in outcome between male and female rats was identified.^26^ In the current investigation, only male rats were used. The protocol for this study, including anesthesia, and the postoperative care of animals was approved by the Institutional Animal Care and Use Committee (IACUC) at Texas A&M University (protocol #2021‐0305‐COD) before the commencement of the study. This study followed the Animal Research Reporting of an In Vivo Experiment (ARRIVE) guidelines.

A sample size of 60 (20 in each group) was determined using previous studies^26^, ^27^ to provide 80% power to identify a mean difference of 0.41 between groups and a standard deviation (σ) of 15% with a 95% confidence interval (α = 0.05).

### 2.2 Study animals

Sixty male Sprague–Dawley rats (*Rattus norvegicus)* with an average weight of 340 g were housed in polycarbonate cages and provided with standard rat chow pellets and water ad libitum. All animals had a 7‐day acclimation period with a 12 h light/12 h dark cycle. Animals were housed in the Animal Research Unit (ARU) following the Animal Welfare Office guidelines.

All animals were checked and weighed daily throughout the experimental period. Animals were randomly assigned to one of three groups using the National Institutes of Health (NIH) randomization tool before the commencement of the study. With the assistance of ARU staff, rat tail markings were used for identification purposes.

The rats were divided into three groups, with each group subjected to a specific topical treatment following surgery:

$$\mathrm{Group}\;1:\;0.12\%\;\mathrm{CHX}\;\left(n\;=\;20\right)$$

$$\mathrm{Group}\;2:\;\mathrm{AO}\;\left(n\;=\;20\right)$$

$$\mathrm{Group}\;3:\;\mathrm{no}\;\mathrm{topical}\;\mathrm{agent}\;\left(n\;=\;20\right)$$

### 2.3 Surgical procedures

**FIGURE 1:**
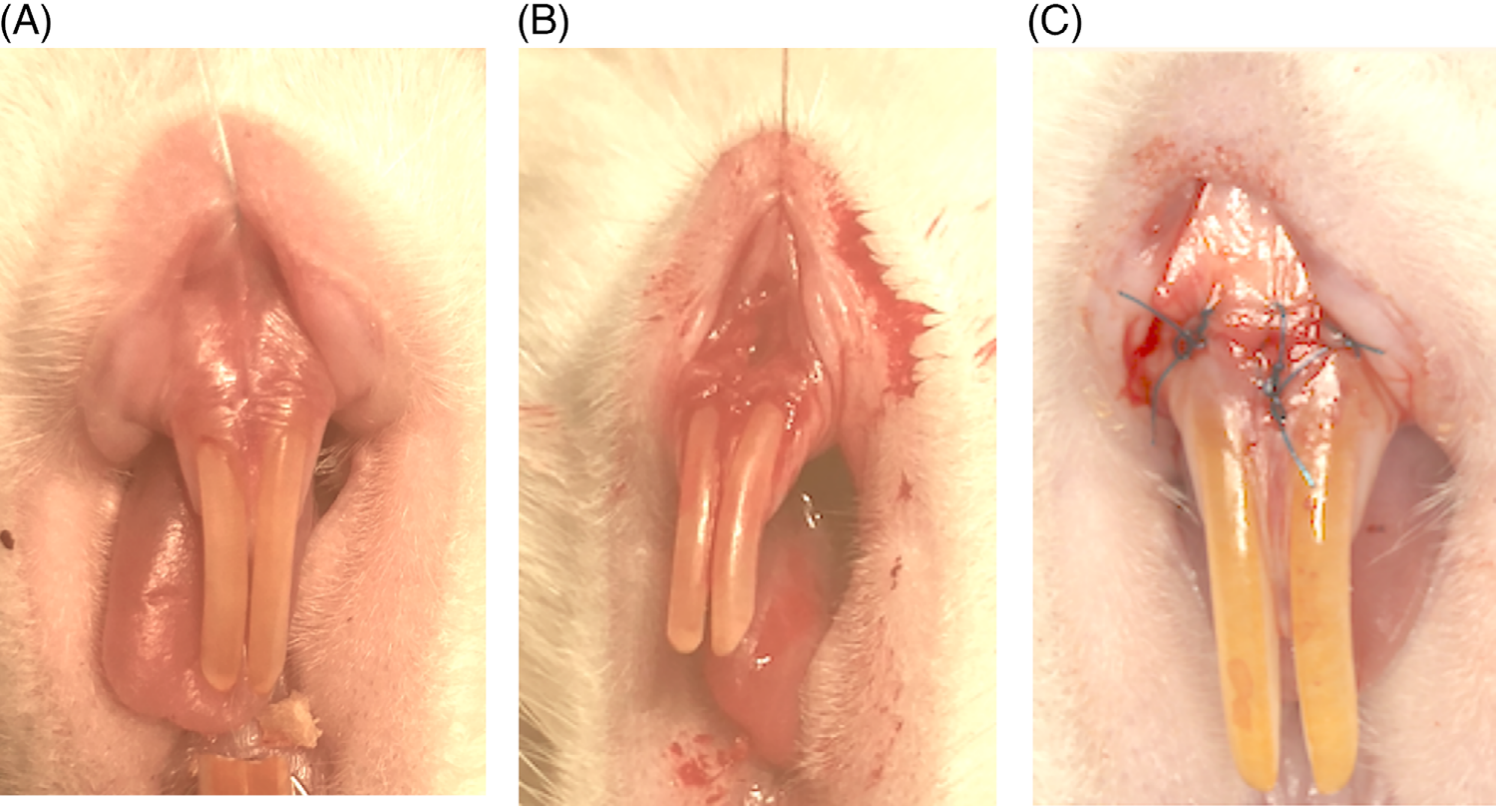
Surgical procedure. (A) Rats were anesthetized and soft tissue retracted. (B) A supraperiosteal pouch was raised before acellular dermal matrix (ADM) insertion. (C) Closure with Prolene sutures.

Before surgeries, rats were weighed and drug doses were adjusted accordingly. General anesthesia was induced with ketamine (0.08 mL/100 g) and xylazine (0.04 mL/100 g) as an intraperitoneal (IP) injection. All animals had the same surgical procedure completed by the same surgeon (K.U.). A microsurgery kit was used to complete the surgeries using a #69 microsurgical mini‐blade† to make the initial submarginal incision in the attached gingiva approximately 5 mm below the gingival margin of the mandibular central incisors, creating a supraperiosteal gingival pouch. ADM was prepared according to the manufacturer's recommendations and hydrated in sterile saline. A 4‐mm‐diameter punch‡ (for standardization) of ADM was placed into the prepared gingival pouch. The surgical site was closed using interrupted 6/0 Prolene sutures§, covering the ADM implant completely (Figure 1). The surgical area was cleaned, and blood was removed with a sterile swab‖.

Rats in Groups 1 and 2 had either one drop of CHX or AO applied to the surgical site with a new sterile swap, respectively.

### 2.4 Postsurgical care

After the operation, rats received a subcutaneous injection of 2–5 mg/kg nalbuphine and were placed under a heating lamp for monitoring.

Rats in Groups 1 and 2 were anesthetized with isoflurane gas for the reapplication of their respective topical agents at least 6 h later on the day of surgery. They continued to be treated twice a day (with at least 6 h between applications) for the duration of the study. The times of application were recorded, and the rats were not fed within 30 min of application.

### 2.5 Study period

Within each treatment group, the animals were divided randomly into two end timepoints (24 and 72 h) post surgery, at which the rats were weighed. The study period was selected as most inflammatory and oxidative stress changes take place in the acute healing phase. The earlier 24‐h timepoint had the following distribution: AO (*n* = 9), CHX (*n* = 8), and control (*n* = 10); these rats were removed from the study. The 72‐h distribution was AO (*n* = 11), CHX (*n* = 11), and control (*n* = 11). Animals were treated in the same order and approximate time, where possible, and were monitored daily for any signs of distress or pain throughout the experimental period.

### 2.6 Specimen recovery procedures

At the postsurgery timepoints allocated, rats were anesthetized with ketamine (0.08 ml/100 mg) and xylazine (0.04 mL/100 g) via IP injections. Tissue specimens were obtained from the surgical site using a 3‐mm‐diameter sterile disposable punch and a sterile microblade. The specimens were frozen at −80°C for mRNA extraction and real‐time polymerase chain reaction (RT‐PCR) analysis.

Samples were also taken for histological processing and stained with hematoxylin and eosin (H&E) (see Supplementary Figure 1 in online *Journal of Periodontology*).

### 2.7 Clinical outcome evaluation

Photographs of the surgical site using a digital single‐lens reflex (SLR) camera with a 100‐mm f/2.8 macro lens¶ were taken of each rat immediately after surgery and at 24 and 72 h depending on their grouping by timepoint. The photographs were numbered, randomized, and presented to 20 double‐blinded scorers who were asked to score the images, as described previously.^26^, ^28^ Individuals scoring were given an initial description of the surgery and shown the appearance of normal healthy rat gingiva (pre‐surgery) and the surgical sites initially post‐surgery. The scorers were also shown examples to score for practice and calibration.

A healing scale was used to assign a healing score from 0 to 8. The assessment included rating gingival color (0—white, 1—partially red, 2—pink), granulation tissue (0—present, 1—not present), epithelization degree (0—not present, 1—present), swelling (0—present, 1—suspected, 2—not present), and graft exposure (0—exposed, 1—indeterminant, 2—not exposed). According to the scale, signs of good healing were tissues that appeared pink with no granulation tissue, swelling, or exposure to the ADM. Poor healing was scored for surgical sites that appeared white and swollen. Due to the short duration of the study, maximum scores (8) were not expected.

### 2.8 Real‐time polymerase chain reaction

The relative expression of TNFα, IL‐1, MPO, and SOD genes was determined using RT‐PCR. The biopsy specimens were stored at −80°C until the RNA was extracted using an RNA extraction kit# to isolate total RNA. The extracted RNA (2 mg) was used to determine the specific genes being expressed in the tissues using sequence‐specific primers (rat IL‐1β Forward 5′ CCAGGATGAGGACCCAAGCA 3′, Reverse 5′ TCCCGACCATTGCTGTTTCC 3′; TNF Forward 5′ AAATGGGCTCCCTCTCATCAGTTC 3′, Reverse 5′ TCTGCTTGGTGGTTTGCTACGAC 3′; MPO Forward 5′ ACCTACCCCAGTACCGATCC 3′, Reverse 5′ AACTCTCCAGCTGGCAAAAA 3′; SOD Forward 5′ ATGTGTCCATTGAAGATCGTGTGA, Reverse 5′ GCTTCCAGCATTTCCAGTCTTTGTA 4′; β‐actin Forward 5′ AGCCATGTACGTAGCCATCC 3′, Reverse 5′ ACCCTCATAGATGGGCACAG 3′; GAPDH Forward 5′ AAGGGCTCATGACCACAGTC 3′, Reverse 5′ GGATGCAGGGATGATGTTCT 3′) using a sequence detection system**. Reaction conditions were 2 min at 50°C (1 cycle), 10 min at 95°C (1 cycle), 15 s at 95°C, and 1 min at 60°C (40 cycles). Samples were normalized to levels of GAPDH or β‐actin. The comparative cycle threshold (CT) method (ΔΔCT) quantified relative differences in mRNA expression. Values are recorded as the mean expression level±  SE.

### 2.9 Percentage weight change

The weight of each rat was taken at baseline (pre‐surgery) and 24 and 72 h post‐surgery and used to calculate the percentage weight change.

### 2.10 Statistical analysis

Data were analyzed using SPSS software version 28.0.1.1. Normally distributed data were analyzed by analysis of variance/analysis of covariance (ANOVA/ANCOVA) and Bonferroni's post hoc testing. For non‐normally distributed data Kruskal–Wallis and Mann–Whitney tests were used. *p* < 0.05 was considered statistically significant.

## 3 RESULTS

### 3.1 Clinical assessment

Clinical scores are summarized in Table 1.

**TABLE 1:** Clinical scores data at 24 h and 72 h.

Parameter	Control	CHX	AO
Overall			
24 h	1.63 ± 0.94	2.03 ± 0.78	3.70 ± 1.28^*^
72 h	1.74 ± 1.06	1.81 ± 1.01	3.24 ± 1.57^*^
Gingival color			
24 h	0.71 ± 0.37	0.62 ± 0.44	1.40 ± 0.40^*^
72 h	0.70 ± 0.39	0.74 ± 0.43	1.34 ± 0.69^*^
Granulation tissue			
24 h	0.01 ± 0.02	0.01 ± 0.02	0.01 ± 0.02
72 h	0.00 ± 0.02	0.01 ± 0.02	0.09 ± 0.02^*^
Epithelization degree			
24 h	0.00 ± 0.00	0.00 ± 0.00	0.00 ± 0.00
72 h	0.00 ± 0.00	0.00 ± 0.00	0.00 ± 0.00
Swelling			
24 h	0.55 ± 0.29	0.69 ± 0.34	0.97 ± 0.37^*^
72 h	0.51 ± 0.34	0.52 ± 0.25	0.95 ± 0.42^*^
Graft exposure			
24 h	0.40 ± 0.39	0.70 ± 0.21	1.09 ± 0.71^*^
72 h	0.63 ± 0.60	0.79 ± 0.43	0.78 ± 0.42

*Note*: Mean ± SD. The AO group scored significantly higher in gingival color, granulation tissue, swelling, and graft exposure compared to CHX and control, respectively. There was no significant difference in epithelization between groups.

Abbreviations: AO, antioxidant gel; CHX, chlorhexidine.

* Denotes score significantly different to CHX and control group (*p* < 0.05).

#### 3.1.1 Overall score

**FIGURE 2:**
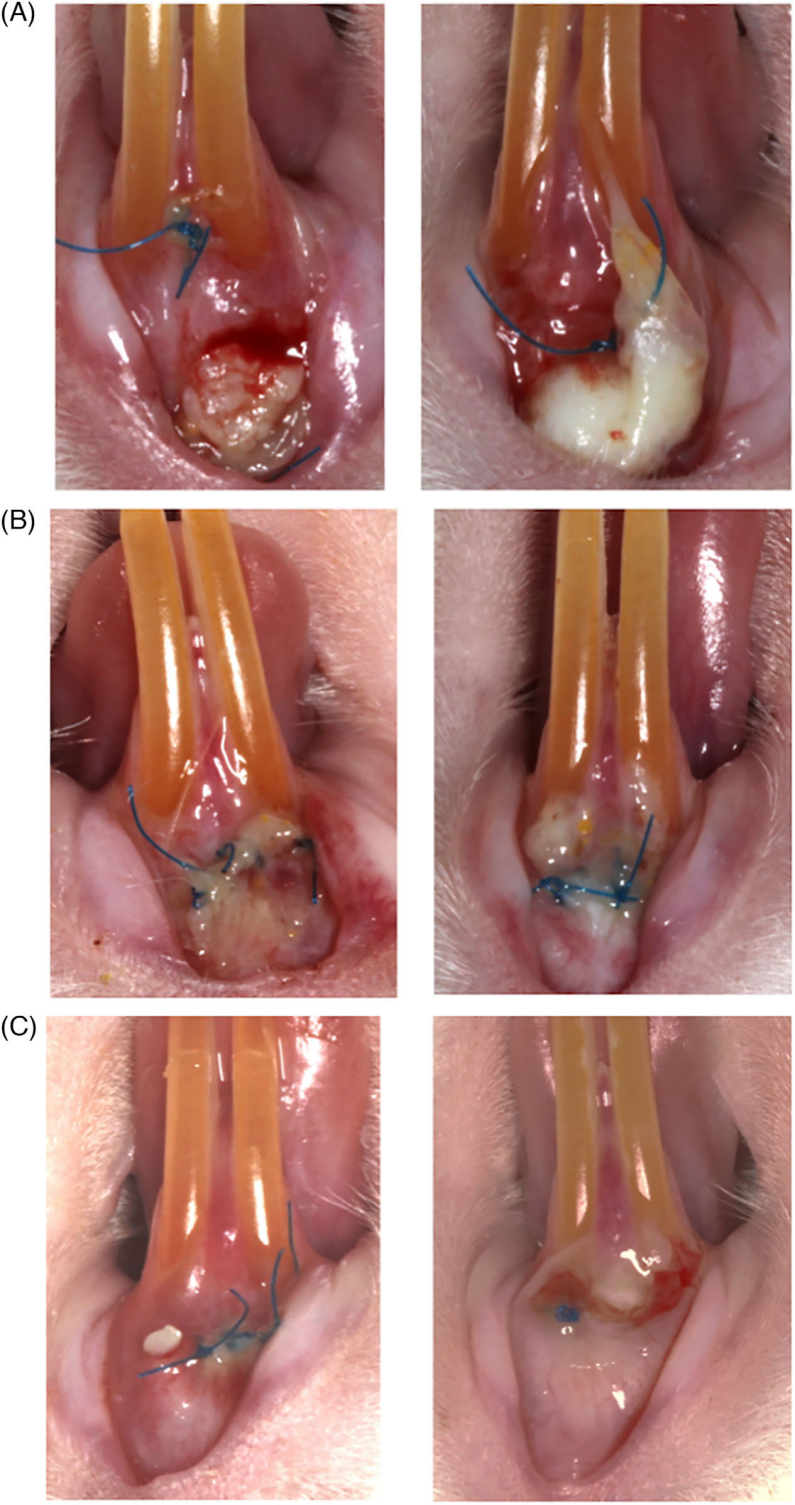
Clinical photos of rats at 24 h (left) and 72 h (right). (A) Control. (B) Chlorhexidine. (C) Antioxidant gel.

There was a significant difference in overall score between the AO group and the other groups, with AO‐treated animals consistently scoring better, indicating better healing outcomes. CHX‐treated animals’ scores were superior to control animals, but this was not statistically significant. The AO‐treated animals’ overall score was significantly higher than the CHX (*p* = 0.009) and control (*p* = 0.001) animals’ after 24 h. Three days later, the AO overall score was still significantly higher than in CHX‐treated (*p* = 0.033) and control‐treated (*p* = 0.024) animals (Figure 2).

#### 3.1.2 Gingival color

The AO group scored significantly higher at both timepoints than CHX and control animal images. CHX animals scored lower than control at 24 h, but the reverse was seen at 72 h, but neither was statistically significant. AO‐treated animals had significantly higher scores for gingival color than CHX (*p* = 0.002) and control (*p* = 0.006) 24 h post surgery. By 72 h post surgery, AO‐treated animals had significantly higher gingival color scores than CHX (*p* = 0.035) and control (*p* = 0.021).

#### 3.1.3 Granulation tissue

At 24 h, there was no significance between the groups. At 72 h, AO scored significantly higher than CHX (*p* = 0.021) and control (*p* = 0.028).

#### 3.1.4 Epithelization degree

There was no statistical difference between the groups regarding the degree of epithelization at the 24‐h or 72‐h timepoints.

#### 3.1.5 Swelling

Overall, AO‐treated animals appeared less swollen than CHX and control animals, as evidenced by a higher score. At 24 h, the AO score was significantly higher than the CHX (*p* = 0.048) and control (*p* = 0.026) score. At 72 h, AO animals had a significantly higher score than CHX (*p* = 0.023) and control (*p* = 0.019) animals and a higher healing score. CHX animals scored marginally higher than control animals at 72 h. However, the difference was not statistically significant.

#### 3.1.6 Graft exposure

AO scored better regarding graft exposure at 24 h. AO animals had a significantly higher score than control (*p* = 0.027) animals, followed by CHX (this was not found to be significant). At 72 h, this was reversed, with CHX‐treated animals having slightly higher scores than AO‐treated animals.

### 3.2 Gene expression analysis

#### 3.2.1 Tumor necrosis factor‐alpha

**FIGURE 3:**
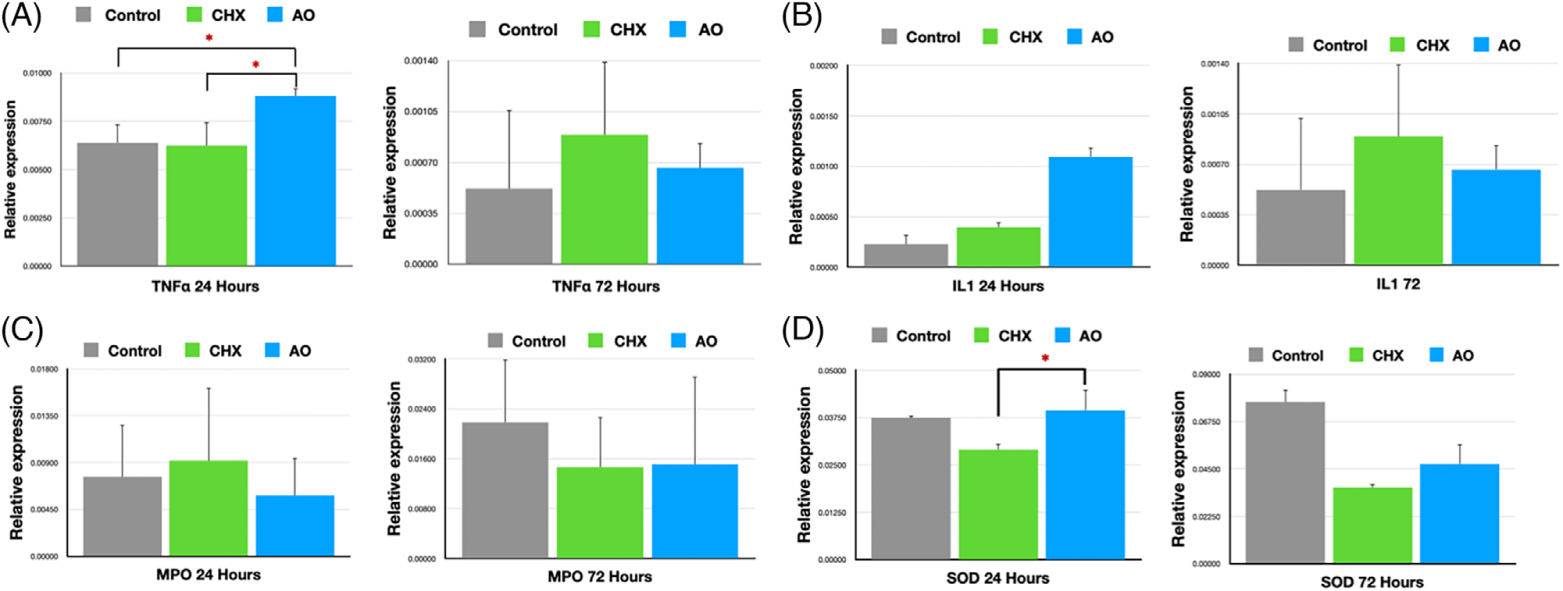
Polymerase chain reaction (PCR) analysis at 24h and 72 h. Mean ± SE. (A) TNFα. (B) IL‐1. (C) MPO. (D) SOD. *p < 0.05. AO, antioxidant gel; CHX, chlorhexidine.

At 24 h, TNFα expression was significantly upregulated in the AO‐treated animals compared to CHX (*p* = 0.027) and control (*p* = 0.018) animals. There was no significant difference in TNFα levels between the CHX and control group. At 72 h, the levels of TNFα expression in the AO‐treated animals decreased, while CHX and control levels increased. CHX animals expressed more TNFα than control animals, but the difference was not statistically significant (Figure 3A).

#### 3.2.2 Interleukin‐1

At 24 h, levels of IL‐1 were highest in the AO group, followed by CHX. This was not statistically significant.

At 72 h, expression decreased in the AO group but increased in the CHX and control groups, with CHX being higher than control. This was not statistically significant (Figure 3B).

#### 3.2.3 Myeloperoxidase

At 24 h, CHX had the highest levels of MPO expression, followed by control. This was not found to be statistically significant. At 72 h, the levels of MPO expression had increased in all three groups, with the highest levels found in control, followed by AO, which was marginally higher than CHX. This was not statistically significant (Figure 3C).

#### 3.2.4 Superoxide dismutase

SOD was significantly upregulated in the AO‐treated animals compared to CHX (*p* = 0.021) in the 24‐h biopsied tissues. The levels of SOD expression in the control animals were higher than in CHX‐treated animals, but it was not statistically significant. At 72 h, there was no statistical difference between the treatment groups (Figure 3D).

### 3.3 Weight change

The mean baseline weight for all animals was 341.19 g, with a common trend of decreasing weight percentage at 24 h and recovery at 72 h for all groups (Table 2). The rats in the CHX group weighed significantly more at baseline compared to the other groups; this was controlled in the statistical analysis with ANCOVA.

**TABLE 2:** Weight change data baseline to 24 h and 24–72 h between the three groups.

Timepoint	Control	CHX^a^	AO
%Δ baseline to 24 h	−2.42	−2.33	−0.13
%Δ 24–72 h	5.93	11.19^b^	7.23^c^

*Note*: The percentage weight of all rats decreased at 24 h and increased at 72 h.

Abbreviations: AO, antioxidant gel; CHX, chlorhexidine.

^a^ There was a significant difference in baseline weight for rats in the CHX group; this was statistically controlled using analysis of covariance (ANCOVA).

^b^ The weight of CHX rats was significantly different to control rats (*p* < 0.05).

^c^ The weight of AO rats was significantly different to control rats (*p* < 0.05).

The mean percentage change between baseline to 24 h was −2.52%, −1.93%, and −0.05% for the control, CHX, and AO groups, respectively. No statistically significant difference was found between the three groups.

At 72 h, analyzing the 33 rats (11 in each group), the mean percentage change between 24 and 72 h was 7.9%, 6.5%, and 10.8% for the control, CHX, and AO groups, respectively. A significant difference in weight change was observed between the AO and control (*p* = 0.034) and CHX and control (*p* = 0.003) groups.

## 4 DISCUSSION

The investigation of periodontal wound healing continues to be significant, running parallel with advancements in postsurgical care strategies. This randomized controlled in vivo study is the first experiment to directly compare AO gel and CHX to nonintervention (control) and demonstrates that AO gel applied topically to the periodontal surgical site may accelerate the healing of tissues and upregulate antioxidant defenses compared to the current gold standard CHX.

Rats are a well‐established animal model and possess anatomy suitable for this investigation. An envelope pouch was made in the lower anterior region. The lower incisor site was chosen as this was the most analogous to humans, although there are some drawbacks, that is, food and bedding can become lodged in the site and this site is more accessible for the rats to use their claws to disturb the wound compared to buccal sites, especially if they are in pain and distress. Rat incisors continue to erupt throughout life which could affect wound stability. However, this risk was considered insignificant as the study period was short.

The surgical technique aimed to simulate the clinical practice of gingival augmentation using ADM. However, there were limitations—passive adaption which would normally be achieved in clinical practice was not possible and would have caused greater variation in inflammation. In addition, ADM in a circular shape rather than linear was selected for standardization during surgery. Due to the tooth anatomy of the rodents, it was not possible to use sling sutures, instead simple interrupted sutures were utilized.

The control group did not have a topical agent applied due to difficulty finding a truly inert agent that would not hydrate or moisturize the tissues, possibly affecting the clinical assessment.^29^ Furthermore, it was crucial to evaluate and compare healing in the absence of any topical intervention. It was appreciated that the treatment agents were in different formulations (AO in gel compared to liquid CHX), which could have affected the outcome. CHX gel was considered; however, it is not currently available for clinical practice in the United States.

The clinical evaluation demonstrated that the AO group scored better than the two other groups overall, suggesting that it could reduce the visible signs of inflammation and promote earlier healing. This finding was apparent for all parameters except epithelization, most likely due to the short length of the study. Statistically, the AO group rats had significantly less graft exposure at 24 h; however, by 72 h, no statistically significant differences were detected. This could be due to tissue shrinkage (reduction in swelling) and the graft not being contained. It was understood that ADM in rats would be less successful than in humans for several reasons, including the rats accessing the surgical site, which could have led to disruption of the sutures and graft. The ADM was 1.6 mm thick, which was adequate to augment human gingiva, which tends to be 0.8–1.5 mm thick.^30^ Rat mucosa is significantly thinner; therefore, exposure of ADM was anticipated.

The gene expression analysis found higher levels of TNFα expression in the AO 24‐h samples compared to the other groups. TNFα functions to recruit leucocytes and phagocytic function. Therefore, we propose that AO promotes earlier neutrophil chemotaxis and wound debridement to the site. An antioxidant agent increasing a proinflammatory response is not unusual. Other investigators have reported that antioxidants such as licorice have an immunomodulatory effect with leucocyte infiltration and angiogenesis.^31^, ^32^ Leucocytes promote ROS release through the respiratory burst, which is beneficial when brief and proportional to the injury. Therefore, antioxidants are required to maintain redox balance as ROS overload may lead to wound breakdown.^33^

SOD is an antioxidant enzyme that decreases the level of ROS superoxide anion. At 24 h, SOD was significantly higher in the AO group compared to CHX, in line with the reports from other investigators, where surgical wounds treated with antioxidant curcumin increased SOD and collagen maturation. ^34^ In this study, the level of SOD was significantly higher in the AO group compared to CHX at 24 h, but not the control, potentially because CHX may decrease fibroblast proliferation early in healing. Fibroblasts have been established as a source of SOD^35^ and therefore have a role in the redox balance. A dose‐dependent relationship between CHX and fibroblast inhibition has been reported causing changes in cell morphology. Adverse effects can be detected as early as 1 min post CHX exposure.^15^ It is likely that the use of the gold standard concentration of CHX, 0.12%, which was used in this study, had deleterious effects on fibroblasts, and CHX‐treated rats would be subject to increased oxidative stress. In contrast, the AO group had efficient scavenging of ROS and earlier inflammation with upregulation of the antioxidant activity.

There was no difference in levels of IL‐1 and MPO between the experimental groups. MPO was not significant between the groups suggesting the neutrophil (O_2_^−^) output was similar for all rats. To fully investigate this gene, rodents in a disease state such as periodontitis^36^ which causes high MPO output would need to be explored. The lack of significance in IL‐1, which similar to TNFα is a proinflammatory marker released early in inflammation, needs to be explored further. This finding contributes to the concept that oral healing is a very complex process. To fully understand the healing process, future experiments should expand on the current research and identify the role of other genes in the cascade. This will require utilizing more genes and techniques to gain a comprehensive understanding of gene interaction in the healing process.

The weights of all rats decreased over the first 24 h, suggesting distress and a change in feeding behavior. Their weight rose again at the 72‐h timepoint, indicating a return to food intake. Applying either topical agent, AO or CHX, appeared to benefit the rats as the two treatment groups had a significant percentage weight recovery compared to the untreated control animals at 72 h. In this study, alterations from the prestudy baseline levels of body weight were used to correlate with distress and reduced water and food intake, similar to other studies.^24^, ^25^ Assessing pain and distress in rodents poses a challenge. The identification of pain in rodents relies on subjective assessments of behavioral and attitudinal shifts, coupled with objective analysis of physiological parameters. Moreover, variations in pain threshold and tolerance must be taken into consideration during this evaluation process.^37^

In a recent study exploring the antibacterial efficacy and its impact on human gingival fibroblasts (HGFs), it was observed that AO gel exhibited a significant level of toxicity toward HGFs.^38^ While HGFs have been essential in dental and periodontal research, many in vitro studies overlook the temporal limitations of HGF survival without fetal bovine serum. Within 6–12 h after nutrient deprivation, HGFs demonstrate signs of cellular distress, leading to reduced viability. This critical period is often associated with cellular stress and apoptosis. Batra et al. lacked a control assessing cell viability at multiple timepoints up to 72 h and did not demonstrate the health of HGF cells at earlier timepoints; it is plausible that cell morbidity was occurring leading up to 72 h. In vitro studies need to be interpreted with care as efforts to mimic cellular interactions may not be accurate.

Further studies are needed to strengthen the current findings, including using AO in other surgical models, such as periodontitis and diabetes. Replication with a more significant number of animals and more gene markers would also be of value.

## 5 CONCLUSION

To the best of our knowledge, this is the first in vivo study to investigate acute inflammation and healing of AO compared to the gold standard CHX and control in an animal model. Within the study's limits, AO demonstrated superior clinical outcomes following gingival augmentation and upregulated antioxidant defenses. Further research is needed to understand the role of topical antioxidants in periodontal wound healing.

## AUTHOR CONTRIBUTIONS

**Kelechi Ukaegbu, BDS, MS**: Completed all surgeries and experiments; set up and analyzed the clinical results; completed gene analysis; compiled the data, and wrote the first draft of the manuscript. **Deborah Foyle, DDS, MS**: Assisted with experimental design; procured funds to support the project, and edited the final manuscript. **Xianghong Luan, BDS, PhD**: Assisted with experimental design and provided the laboratory equipment and expertise for gene analysis. **Emet Schneiderman, PhD**: Assisted with statistical design and edited the manuscript. **Edward P. Allen, DDS, MS, PhD**: Assisted in drafting and editing the manuscript. **Jacqueline Plemons, DDS, MS**: Assisted with experimental design and edited the manuscript. **Kathy K.H. Svoboda, PhD**: Designed the experiments including data acquisition; data analysis, and figure production and edited the manuscript.

## CONFLICT OF INTEREST STATEMENT

The authors declare no conflicts of interest.

## Supporting information

Supporting Information

## Footnotes

* AlloDerm Biohorizons, Birmingham, Alabama, USA.

† Salvin Dental Specialties, Charlotte, North Carolina, USA.

‡ Premier Uni‐Punch, Plymouth Meeting, Pennsylvania, USA.

§ Hu‐Friedy, Chicago, Illinois, USA.

‖ McKesson, Irving, Texas, USA.

¶ Canon, Taichung City, Taiwan.

# RNeasyPlus Mini Kit; Qiagen, Venlo, The Netherlands.

** SYBR green Master Mix and the ABI Prism 7000 Sequence detection system; Applied Biosystems, Thermo Fisher Scientific, Waltham, Massachusetts, USA.
