# Supplementary material for: The effect of an antioxidant gel compared to chlorhexidine during the soft tissue healing process: An animal study
Source: J Periodontol. 2024 Jun 3;95(11):1086–96. doi: 10.1002/JPER.23-0794 (PMC11609495; doi:10.1002/JPER.23-0794)
Supplement: Supplementary file 1 — Supporting Information [file JPER-95-1086-s001.docx]

Supplementary Figure Legend

Figure 1. Histological specimens of the three groups control, CHX, and AO at 24 and 72 hours respectively. All slides show mild to moderate inflammation in the lamina propria, with no discernible differences between groups
